# Type-II Band Alignment and Tunable Optical Absorption in MoSSe/InS van der Waals Heterostructure

**DOI:** 10.3389/fchem.2022.861838

**Published:** 2022-02-22

**Authors:** X. B. Yuan, Y. H. Guo, J. L. Wang, G. C. Hu, J. F. Ren, X. W. Zhao

**Affiliations:** ^1^ School of Physics and Electronics, Shandong Normal University, Jinan, China; ^2^ Shandong Provincial Engineering and Technical Center of Light Manipulations and Institute of Materials and Clean Energy, Shandong Normal University, Jinan, China

**Keywords:** van der waals heterostructure, first principles calculations, optical absorption, biaxial strain, band edge position

## Abstract

In this work, we study the electronic structure, the effective mass, and the optical properties of the MoSSe/InS van der Waals heterostructures (vdWHs) by first-principles calculations. The results indicate that the MoSSe/InS vdWH is an indirect band gap semiconductor and has type-Ⅱ band alignment in which the electrons and holes located at the InS and the MoSSe side, respectively. The band edge position, the band gap and the optical absorption of the MoSSe/InS vdWH can be tuned when biaxial strains are applied. In addition, compared with MoSSe and InS monolayers, the optical absorption of the MoSSe/InS vdWH is improved both in the visible and the ultraviolet regions. These findings indicate that the MoSSe/InS vdWHs have potential applications in optoelectronic devices.

## Introduction

With the discovery of graphene in 2004, two-dimensional (2D) materials have been widely studied and applied due to their unique structures and excellent physical and chemical properties (Novoselov et al., 2004). In the past decades, a large number of 2D materials have been emerged, such as transition metal dichalcogenides (TMDCs) (Choi et al., 2017; Qiu et al., 2020), silicene (Fleurence et al., 2012; Wu et al., 2014), group-III monochalcogenides (Huang et al., 2019), Mxenes (Zhou et al., 2021), etc. In order to obtain high-performance devices, the advantages of a single layer 2D material are slightly insufficient, so the van der Waals heterostructures (vdWHs) formed by the van der Waals forces along the vertical superposition of two different 2D materials has been attracted widespread attention (Guo et al., 2021a; Zhao et al., 2021). A variety of vdWHs have been developed, which can be classified into three band alignments, i.e., straddling type-I, staggered type-II, and broken-gap type-III (Özçelik et al., 2016).

The vdWHs not only contain some of the advantages of the isolated monolayers but also produce some other properties (Ren et al., 2021a; Ren et al., 2021b; Ren et al., 2021c; Sun et al., 2022; Shen et al., 2022). For example, Liu *et al.* proposed the MoSSe/g-GeC heterostructure as a promising photovoltaic application material in which visible optical absorption and catalytic activity can be adjusted by strain engineering (Liu et al., 2021). M. M. Obeid and others revealed that GaSe/HfS_2_ heterostructures have high carrier mobility and can be converted from semiconductor to metal and from indirect band gap to direct band gap when the external electric field is strengthened (Obeid et al., 2020). Zhu *et al.* found that GaN/Zr_2_CO_2_ heterostructure has a promising application in tunable high-performance optoelectronic nanodevices due to its large conduction band offset (CBO) and tunable band gap (Zhu et al., 2021). Zhang *et al.* proved that P-GaSe/InS isomorphous heterostructure has excellent performance as a photocatalytic and water splitting material (Zhang et al., 2021).

On the other hand, after Janus MoSSe was successfully synthesized by chemical vapor deposition (CVD) method in 2017 (Lu et al., 2017; Zhang et al., 2017), the unique physical properties of 2D Janus TMDCs due to their mirror asymmetry have been attracted widespread attention. The Janus MoSSe has an easy-to-tune band gap, strong visible optical absorption and suitable band alignment (Yin et al., 2018); Mo atoms are located between S atoms and Se atoms, so an internal electric field is formed, which promotes electron-hole separation and inhibits exciton recombination (Chen et al., 2019). In addition, as a new type of 2D material, Group-III chalcogenides (MX, M = Ga and In, X = S, Se) have been attracted great attention in photoelectric devices due to their wide band gap, high electron mobility, good thermoelectric performance and optical responses (Miao et al., 2016; Xu et al., 2016; Hung et al., 2017). InS has been successfully synthesized experimentally and has a similar structure to InSe (Hollingsworth et al., 2000). Monolayer InS has good optical response characteristics and large band gap. However, InS has some disadvantages in visible optical absorption due to the large indirect band gap, and the separation ability of electron-hole pairs is also weak. Both MoSSe and InS monolayers can be synthesized, so the MoSSe/InS vdWH is also feasible composed experimentally. Therefore, we hope that the MoSSe/InS vdWH has the advantages of the two monolayers.

In this work, based on the first-principles calculations, we mainly study the band edge position, the charge transfer and the optical absorption of the MoSSe/InS vdWH. Effects of biaxial strain on electronic structure and optical properties are also considered. Compared with MoSSe and InS monolayers, the optical absorption of the MoSSe/InS vdWH is improved and can be modulated by biaxial strain. The structure of this paper is as follows: details of the computational methods are provided in Section 2, the results and the discussion are shown in Section 3, and the conclusion is presented in Section 4.

## Computational Methods

All first-principles calculations are based on density functional theory (DFT) by using Vienna ab initio Simulation Package (VASP) (Kresse and Furthmüller, 1996). The core-ion and valence electron interaction is described by the projector augmented wave (PAW) method, and the general gradient approximation (GGA) in the form of Perdew-Burke-Ernzerhof (PBE) functional is used to calculate the exchange-correlation functional (Blöchl, 1994; Perdew et al., 1996; Kresse and Joubert, 1999). For the plane-wave basis set, the energy cutoff is set to 500 eV. All geometric structures are completely relaxed with the total energy is converged within $1\times 10^{-6}\;$ eV and the Hellmann–Feynman force is less than 0.01 eV ${Å}^{-1}$ . In the empirical correction scheme proposed by Grimme, we use the DFT-D3 method to describe the effect of the interaction between monolayers (Grimme et al., 2010; Grimme et al., 2011). The k-points in the first Brillouin-zone of 4 × 4 × 1 and 8 × 8 × 1 are generated by the Monkhorste Packscheme and they are used for the geometric optimization and the self-consistent calculations, respectively. To avoid the interaction caused by periodic effects, the vacuum layer is set to 20 Å along the *Z*-axis direction. Since the PBE always underestimates the band gap of the semiconductor, we use hybrid functional (HSE06) to further obtain accurate electronic properties (Heyd et al., 2003). The VASP processing program VASPKIT is used to analyze electronic structure and optical properties (Wang et al., 2021).

## Results and Discussion

**FIGURE 1:**
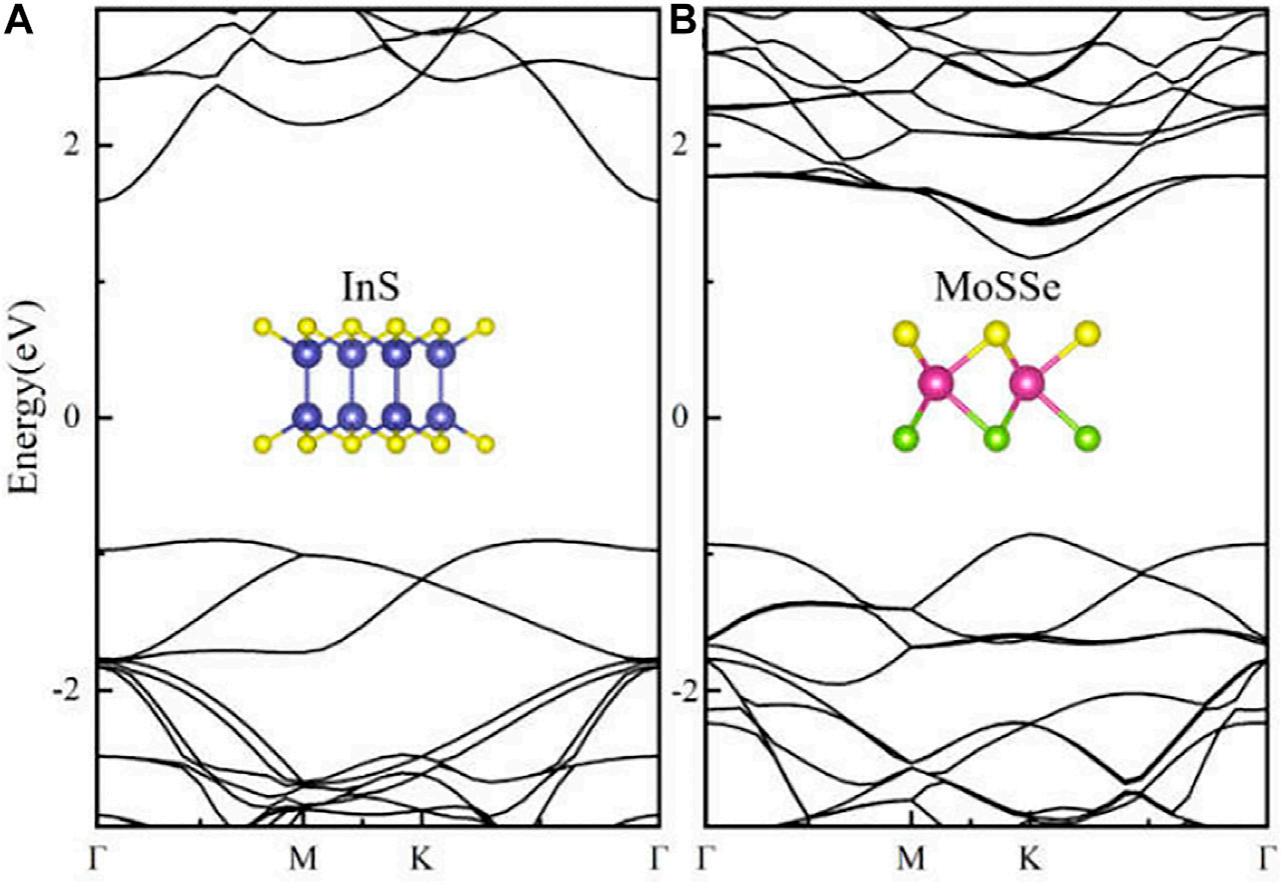
Band structures of **(A)** InS and **(B)** MoSSe monolayers.

Before investigating the MoSSe/InS vdWH, the structural parameters and the electronic characteristics of the MoSSe and InS are studied. The optimized lattice parameters of the MoSSe and InS monolayers are a = b = 3.25 Å and a = b = 3.94 Å respectively. The results are consistent with the previous reported results (Zhu et al., 2021; Hollingsworth et al., 2000; Guo et al., 2021b). Taking into account the lattice mismatch, we constructed a 2 × 2 MoSSe supercell and a √3×√3 InS supercell to form the MoSSe/InS vdWH to achieve a small lattice mismatch value of 4.8%. The calculated electronic band structures of the monolayers by HSE06 functional are plotted in Figure 1. We can find that the InS and the MoSSe have indirect band gap and direct band gap, respectively. In Figure 1A, the band gap value of InS is 2.48 eV. Its conduction band minimum (CBM) is located at the Γ point, and its valence band maximum (VBM) is located between the Γ point and the M point. As shown in Figure 1B, the band gap value of MoSSe is 2.03 eV. Its CBM and VBM are both located at the K point. These results of the MoSSe and InS monolayers are consistent well with previous results (Hollingsworth et al., 2000; Zhu et al., 2021).

**FIGURE 2:**
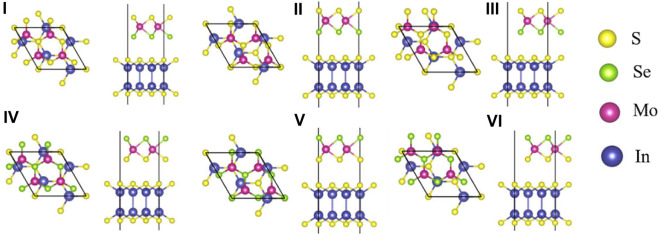
The top and side views of six stacking patterns of the MoSSe/InS vdWHs. The yellow, green, purple and blue balls represent the S, Se, Mo and In atoms, respectively.

For the MoSSe/InS vdWH, after considering the different stacking patterns between the layers, there are six typical stacking configurations, see Figure 2. Among them, I-III correspond to the cases when Se atoms are adjacent to InS monolayer, IV-VI correspond to the cases when S atoms are adjacent to InS monolayer. In order to compare the stability of the six configurations, their binding energies are calculated according to the formula: $E_{b}=E_{total}-E_{MoSSe}-E_{InS}$ , where $E_{total}$ , $E_{MoSSe}$ , and $E_{InS}$ are the total energy of the MoSSe/InS vdWH, the energy of the MoSSe monolayer and the energy of the InS monolayer, respectively. The calculated binding energies of the I-VI configurations are −4.078 meV, −4.083 meV, −4.073 meV, −4.057 meV, −4.054 meV, −4.050 meV, respectively. All the six configurations show negative binding energies, which indicate that the MoSSe/InS vdWH is thermodynamically stable. Therefore, the II stacking pattern with the smallest binding energy is considered as the most stable one and it is used in the following calculations.

**FIGURE 3:**
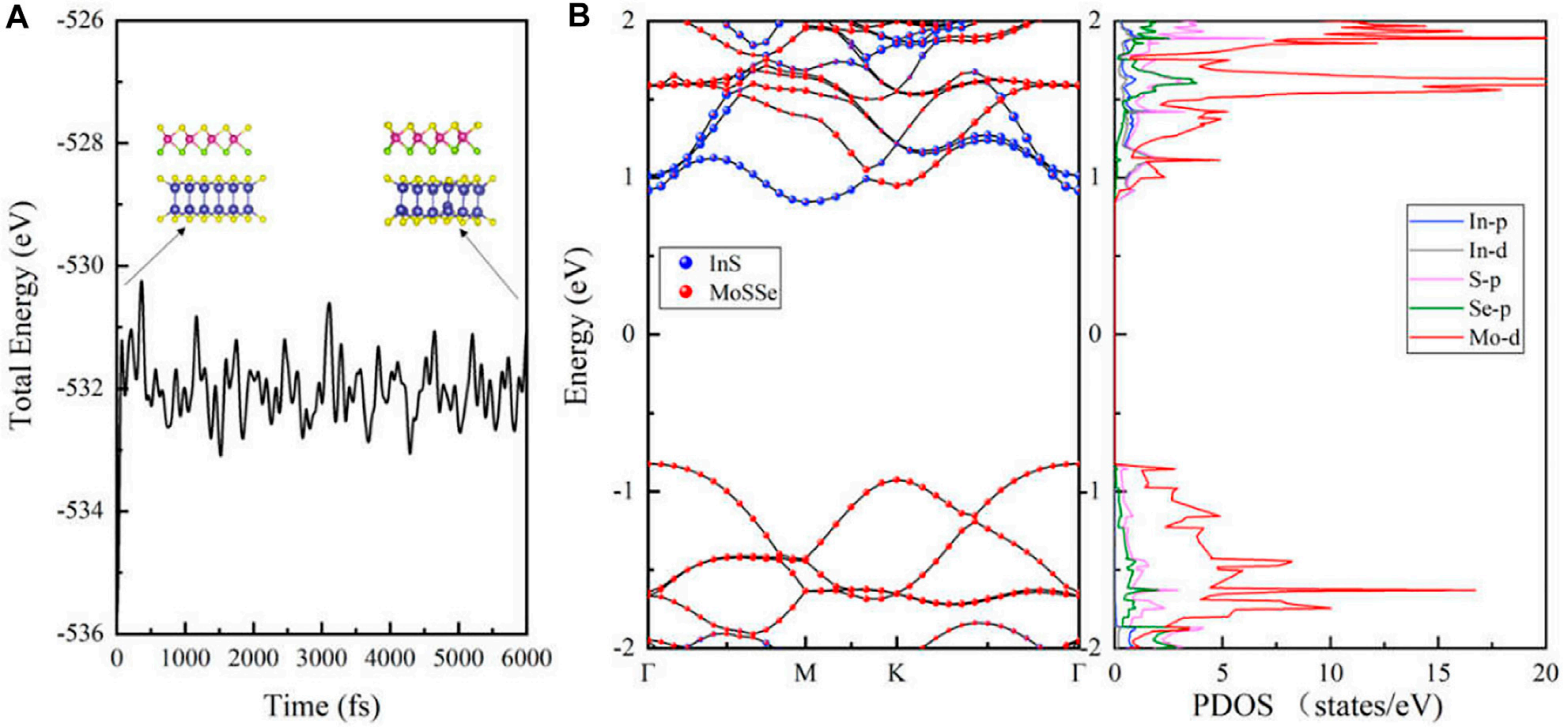
**(A)** The total energy and the crystal structures calculated from the AIMD of the MoSSe/InSe vdWH. **(B)** The projected band structures and the PDOS of the MoSSe/InS vdWH.

In order to verify the thermodynamical stability, we perform ab initio molecular dynamics (AIMD) calculations for the MoSSe/InS vdWH at 300 K, as shown in Figure 3A. The simulations last for 3 ps with a time step of 0.5 fs. In the calculation of AIMD, the energy fluctuation of the vdWH is very small and the structure has no distortion, indicating that the vdWH has good thermal stability.

Figure 3B shows the projected band structure and the projected density of states (PDOS) of the MoSSe/InS vdWH calculated by HSE06 functional. In Figure 3B, it can be seen that the MoSSe/InS vdWH is an indirect band gap semiconductor with a band gap value of 1.67 eV, which is slightly smaller than those of MoSSe and InS monolayers and larger than the redox potential energy of water (1.23 eV). In addition, its CBM and VBM are located at M point and Γ point respectively, and it has type-Ⅱ band alignment because its CBM and VBM are contributed from InS and MoSSe monolayers respectively. The type-Ⅱ band alignment can effectively promote the spontaneous separation of electron-hole pairs, making it applicable to various photocatalytic and solar energy conversion devices (Massicotte et al., 2016; Lei et al., 2019). The PDOS also proves our suggestion. It can be seen that the CBM is mainly contributed from the In-p, In-d and S-p orbitals, and the VBM is mainly contributed from the Mo-d orbitals.

**FIGURE 4:**
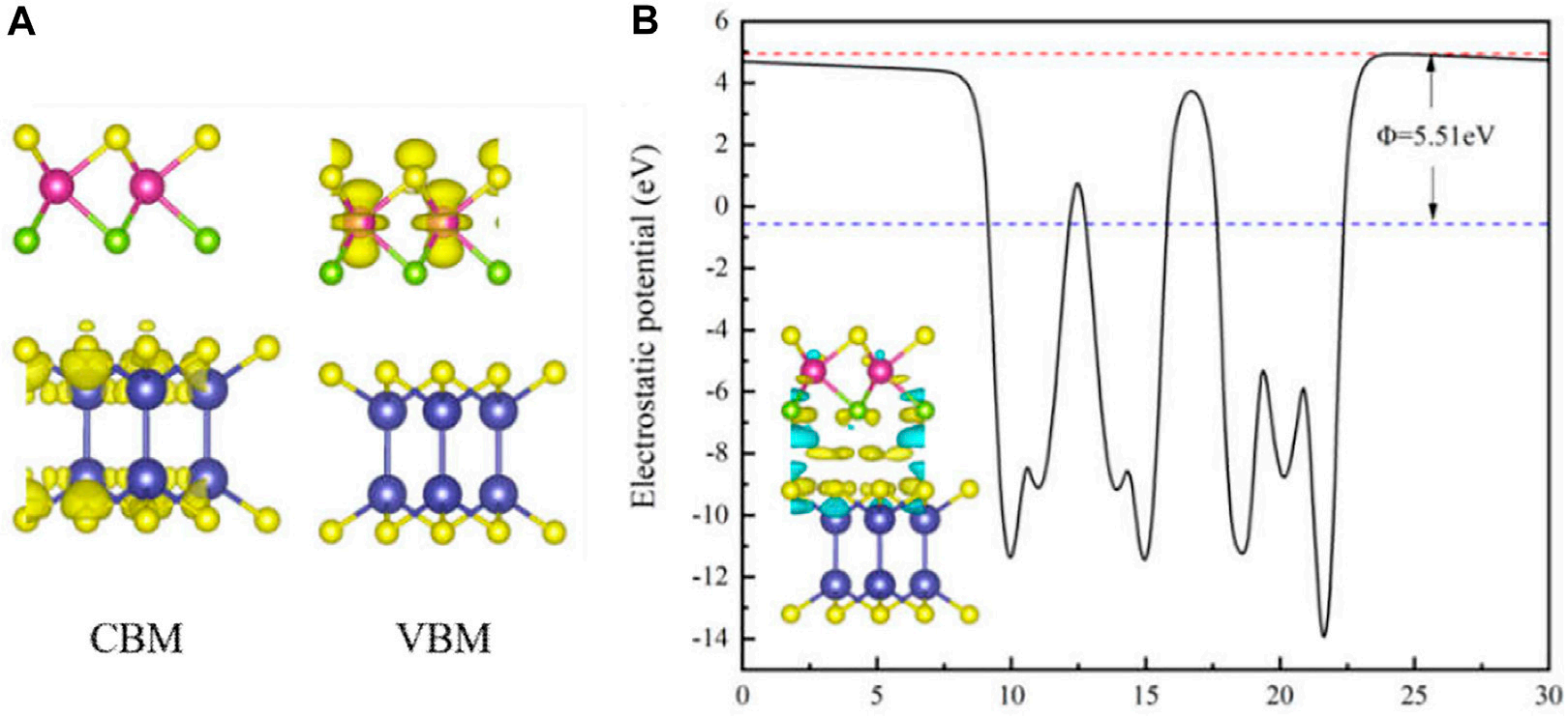
**(A)** The band decomposition charge densities of CBM and VBM for the MoSSe/InSe vdWH. **(B)** The electrostatic potential and the charge density difference of the MoSSe/InS vdWH.

As shown in Figure 4A, the band decomposition charge densities of CBM and VBM are calculated. CBM and VBM are concentrated in the InS and MoSSe layers respectively, which means that the electrons are located in the InS layer and the holes are located in the MoSSe layer, respectively. In order to further understand the charge transfer mechanism between MoSSe and InS, we calculate the electrostatic potential and the charge density differences of the MoSSe/InS vdWH, as shown in Figure 4B. The red dotted line and the blue dotted line represent the vacuum level (E_vac_) and the Fermi level (E_f_), respectively, and Φ is the work function which can be obtained by the difference between Evac and E_f_, and its value is 5.51 eV. The charge density difference is in the lower left corner of Figure 4B, which is given by $\Delta \text{ρ}=\rho_{M\text{oSSe}/\text{InS}}-\rho_{MoSSe}-\rho_{InS}$ , where $\rho_{MoSSe/InS}$ , $\rho_{MoSSe}$ and $\rho_{InS}$ are the total electron densities of the MoSSe/InS vdWH, MoSSe monolayer and InS monolayer, respectively. The yellow represents charge accumulation and the blue represents charge depletion. The calculated results show that the electrons are mainly concentrated on the InS side, while the holes are concentrated on the MoSSe side, so the electrons transfer from the MoSSe side to the InS side. We also calculate the effective mass of the electron along different directions according to the formula: $m^{*}=\pm \hbar^{2}{\left(\frac{d^{2}E_{k}}{dk^{2}}\right)}^{-1}$ . The effective masses of electrons ( $m_{e}^{\text{*}}$ ) are 0.569 from K to M point and 0.338 from Γ to M point.

**FIGURE 5:**
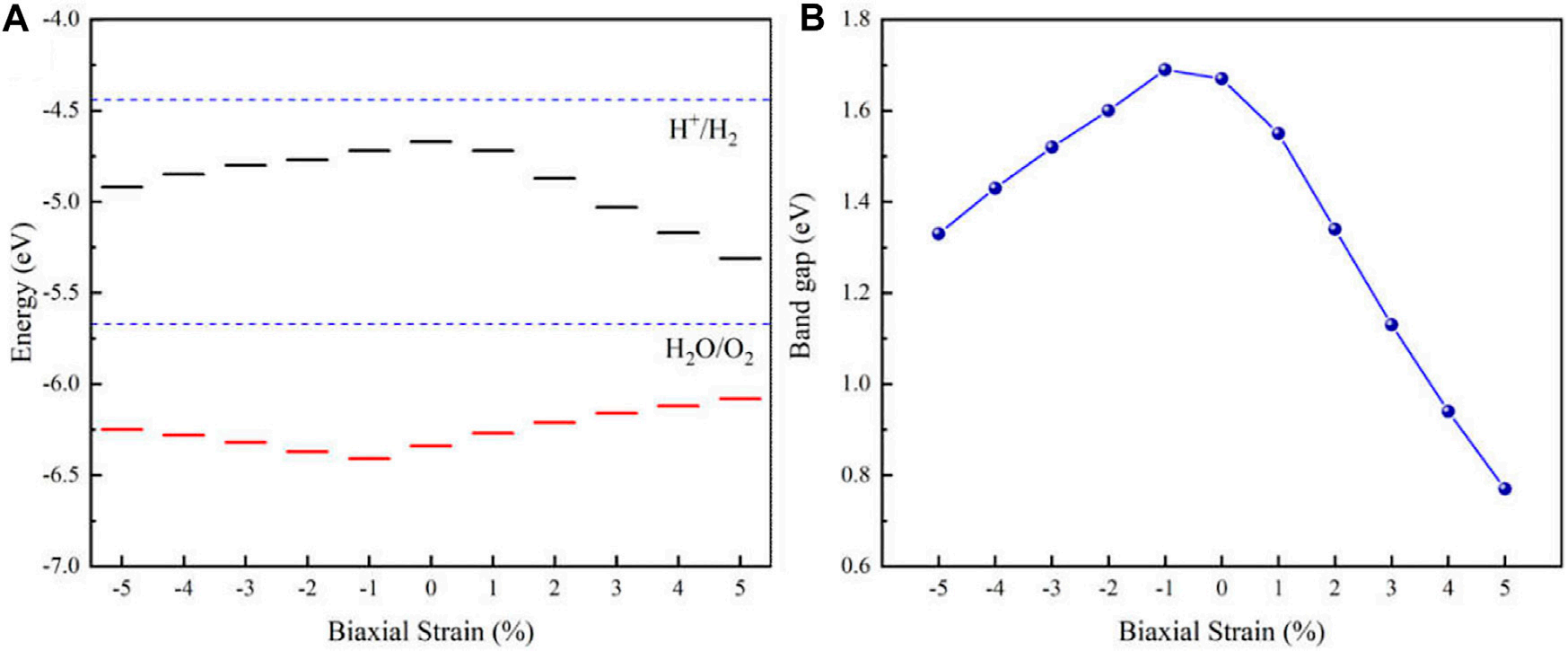
**(A)** Band edge positions and **(B)** band gap of the MoSSe/InS vdWH under various biaxial strains from −5 to 5%.

2D materials can withstand greater strain than three-dimensional materials, so biaxial strain is a method that can effectively modulate the electronic structure and the optical properties of 2D materials (Guo et al., 2020; Zhu et al., 2021). In this paper, we study the effect of biaxial strain on the band gap, band edge position and the optical absorption of the MoSSe/InS vdWH. The biaxial strain can be defined as *ε*= (a−a_0_)/a_0_×100%, where a_0_ and a are the lattice constants of the strained-free and strained structures, correspondingly. In Figure 5A, the blue dashed line marks the redox potentials of H_2_O/O_2_ (−5.67 eV) and H^+^/H_2_ (−4.44 eV) of water. It can be seen that both the unstrained and strained band edges of the MoSSe/InS vdWH cross the oxidation potential of H_2_O/O_2_, so they are suitable for driving oxygen evolution reaction kinetics when pH = 0. In addition, when the applied pressure and tension increase, the energy levels of the CBM and the VBM move down and up, respectively. Figure 5B illustrates that the band gap of the MoSSe/InS vdWH decreases when the tensile strain increase, however, when the compressive strain increases, the band gap firstly reaches to a maximum and then decrease. This tunable band gap has potential applications in devices.

**FIGURE 6:**
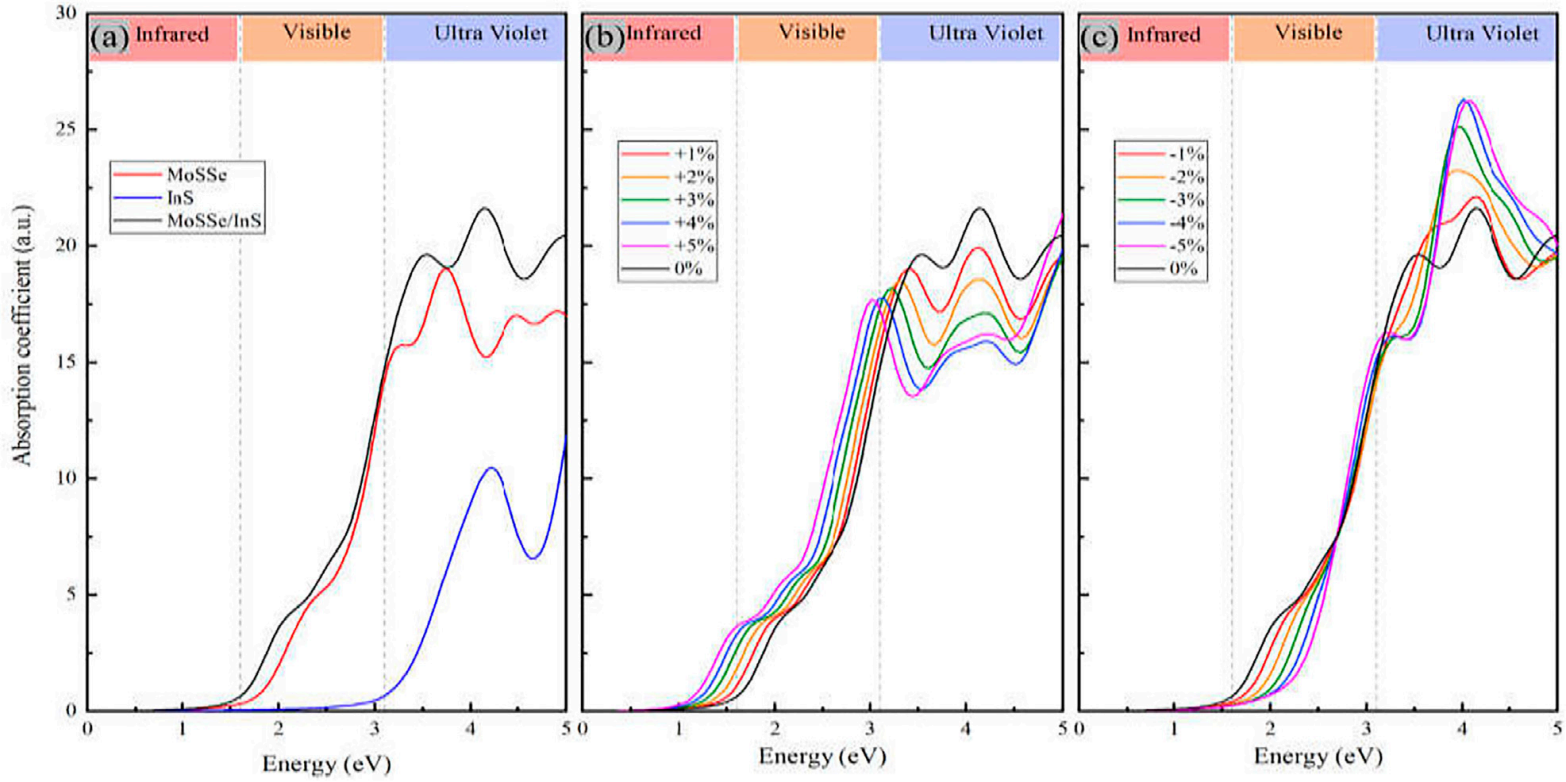
**(A)** The optical absorption spectra of MoSSe monolayer, InS monolayer and the MoSSe/InS vdWHs, **(B,C)** correspond to the cases with tension strain and compressive strain, respectively.

The optical absorption is calculated by the formula,

$$\alpha \left(\omega \right)=\frac{\sqrt{2}\omega }{c}{\left\{{\left[\varepsilon_{1}^{2}\left(\omega \right)+\varepsilon_{2}^{2}\left(\omega \right)\right]}^{\frac{1}{2}}-\varepsilon_{1}\left(\omega \right)\right\}}^{\frac{1}{2}}$$ (1)

where ω is the light frequency, $\varepsilon_{1}\left(\omega \right)$ and $\varepsilon_{2}\left(\omega \right)$ represent the real and the imaginary parts of the complex dielectric function, respectively. Figure 6A gives the optical absorption spectra of the MoSSe/InS vdWH and the two independent monolayers, and (b) and (c) correspond to the cases under +1%∼+5% tension strain and −1%∼−5% compressive strain, respectively. As shown in Figure 6A, InS has a weaker optical absorption compared with that of the MoSSe. On the other hand, optical absorption of InS in the ultraviolet region is stronger than that in the visible light region. As a good photocatalytic material, MoSSe has a very strong optical absorption. Charge transfer and interlayer coupling promote the overlap of orbitals in the heterostructure, so the MoSSe/InS vdWH exhibits a stronger light absorption capacity compared with MoSSe and InS monolayers. Strain engineering is an important method to modulating the properties of 2D materials. We apply biaxial strain to study the optical properties of the MoSSe/InS vdWH. In Figure 6B, it can be found that the optical absorption of the MoSSe/InS vdWH in the visible light region increases when the tension strain increases, while in the ultraviolet region it decreases when the tension strain increases. As shown in Figure 6C, when the compressive strain increases, the optical absorption of the MoSSe/InS vdWH exhibits the opposite phenomenon compared with the case when tension strain is applied.

## Conclusion

In summary, we have explored the electronic structure and the optical properties of the MoSSe/InS vdWH by first-principles calculations. Our results show that the MoSSe/InS vdWH has an indirect band gap with typical type-II band alignment that can effectively promote the spontaneous separation of electron-hole pairs. CBM and VBM are contributed from InS and MoSSe monolayers respectively, and electrons are transferred from the MoSSe layer into the InS layer in MoSSe/InS vdWH. Compared with monolayers, the carrier mobility and the optical absorption of the vdWH are enhanced. After the application of biaxial strain, the position of the band edge is adjusted, and the band gap of the vdWH can also be tuned. In the visible light region, the optical absorption intensity of the MoSSe/InS vdWH increases with the increasing of the tensile strain, and it decreases with the increasing of the compressive strain. Our work shows that the MoSSe/InS vdWH may have potential applications in optoelectronic devices.

## Data Availability

The original contributions presented in the study are included in the article/Supplementary Material, further inquiries can be directed to the corresponding authors.
